# Early initiation of a strength training based rehabilitation after lumbar spine fusion improves core muscle strength: a randomized controlled trial

**DOI:** 10.1186/s13018-018-0853-7

**Published:** 2018-06-19

**Authors:** Dejan Kernc, Vojko Strojnik, Rok Vengust

**Affiliations:** 1 Faculty of Sport, University of Ljubljana, Gortanova 22, 1000 Ljubljana, Slovenia; 2 Faculty of Medicine, University of Ljubljana, Vrazov trg 2, 1000 Ljubljana, Slovenia

**Keywords:** Rehabilitation, Lumbar spine fusion, Randomized controlled trial, Strength training, Early initiation, Intra-abdominal pressure

## Abstract

**Background:**

To analyze the safety and effects of early initiation of rehabilitation including objective measurement outcomes after lumbar spine fusion based on principles of strength training.

**Methods:**

The study recruited 27 patients, aged 45 to 70 years, who had undergone lumbar spine fusion. The method of concealed random allocation without blocking was used to form two groups. The strength training group started rehabilitation 3 weeks after surgery. Patients exercised twice weekly over 9 weeks focusing on muscle activation of lumbopelvic stabilization muscles. The control group followed a standard postoperative protocol, where no exercises were performed at that stage of rehabilitation. Functional outcomes and plain radiographs were evaluated at 3 weeks and subsequently at 3 and 18 months after the surgery.

**Results:**

No hardware loosening of failure was observed in the training group. Both groups improved their walking speed after 3 months (*p* < 0.01), although improvement in the training group was significantly greater than in the control group (*p* < 0.01). Moreover, the training group significantly improved after the training period in all isometric trunk muscles measurements (*p* < 0.03), standing reach height (*p* < 0.02), and pre-activation pattern (*p* < 0.05). After 18 months, no training effects were observed.

**Conclusions:**

The study showed that early initiation of a postoperative rehabilitation program based on principles of strength training is safe, 3 weeks after lumbar spine fusion, and enable earlier functional recovery than standard rehabilitation protocol.

**Trial registration:**

The study is registered at the US National Institutes of Health (ClinicalTrials.gov) NCT03349580. The date of registration: November 21, 2017 - Retrospectively registered.

## Background

Despite the significant rise in lumbar spine fusion (LSF) surgery rates in the last few decades, some 15 to 40% of lumbar fusion patients cannot expect significant improvement postoperatively according to functional ability [1–6]. The postoperative rehabilitation strategy is one of the main factors affecting the outcome. Nevertheless, only a few studies address the effect of different protocols and timing of postoperative rehabilitation [7, 8].

The benefit of intra-abdominal pressure (IAP) on lumbar spine function is well documented [9, 10]. IAP with co-activation of the abdominal muscles provides load relief to the lumbar spine and increased stability of the trunk [10, 11]; however, IAP needs to start rising before the initiation of action to have a protective effect on the lumbar spine [12].

There is some disagreement on the optimal time to initiate a rehabilitation program after LSF. A randomized controlled trial from 2013 evaluating the impact of initiating rehabilitation either 6 or 12 weeks after LSF demonstrated no difference to the patient’s physical performance in terms of fitness and walking distance [8]. A study published in 2014 showed that early initiation of rehabilitation does not increase the risk of postoperative complications [13]. Conversely, Oestergaard et al. [14] showed that initiating rehabilitation after 12 weeks resulted in a significantly better clinical improvement compared to an earlier initiation.

Rehabilitation after LSF aims to improve the trunk muscles’ functional capacity [7, 15]. Evaluation of LSF rehabilitation protocols should include, in addition to subjective outcomes (ODI), an objective measurement of functional ability such as strength of the stabilization muscles, physical performance, etc.

Given the above, the aim of the present study was to analyze the safety and effects in the early initiation of strength training that promote trunk stabilization through IAP and utilizing both subjective and measurable objective outcomes.

## Methods

### Study design, selection of subjects, surgery, and follow-up

The study was a randomized controlled trial with a baseline measurement at 3 weeks after LSF, and additionally at 3 and 18 months after LSF. The selection of subjects and surgeries were determined by three consultant spine surgeons at the Slovenian national spine center in Ljubljana.

The subjects were recruited over a 14-month period (September 2014–November 2015). The inclusion criteria for subjects were (1) a primary diagnosis of one level degenerative, low-grade isthmic spondylolisthesis or degenerative disc disease, with or without spinal stenosis; (2) age between 45 and 70 years; and (3) the absence of non-communicable diseases. The exclusion criteria for subjects were (1) previous lumbar fusion surgery, (2) degenerative or idiopathic scoliosis, (3) and inflammatory disease and history of malignancy. The National Medical Ethics Committee approved the study.

Subjects had received one level instrumented transforaminal interbody fusion. A cage of maximal feasible height was placed as anteriorly as possible to obtain segmental lordosis. Decompression was employed with respect to primary pathology, central/lateral recess stenosis in degenerative spondylolisthesis, and foramina in isthmic spondylolisthesis. No decompression was performed in patients with degenerative disc disease.

Additional control checks were employed to ensure subject safety in the exercise protocol, and subjects were examined by the managing surgeon. At 2 and 18 months postoperatively, plain radiographs were taken. Furthermore, at 18 months postoperatively, flexion/extension films were obtained to rule out hardware loosening or failure.

### Sample size and randomization

**Fig. 1:**
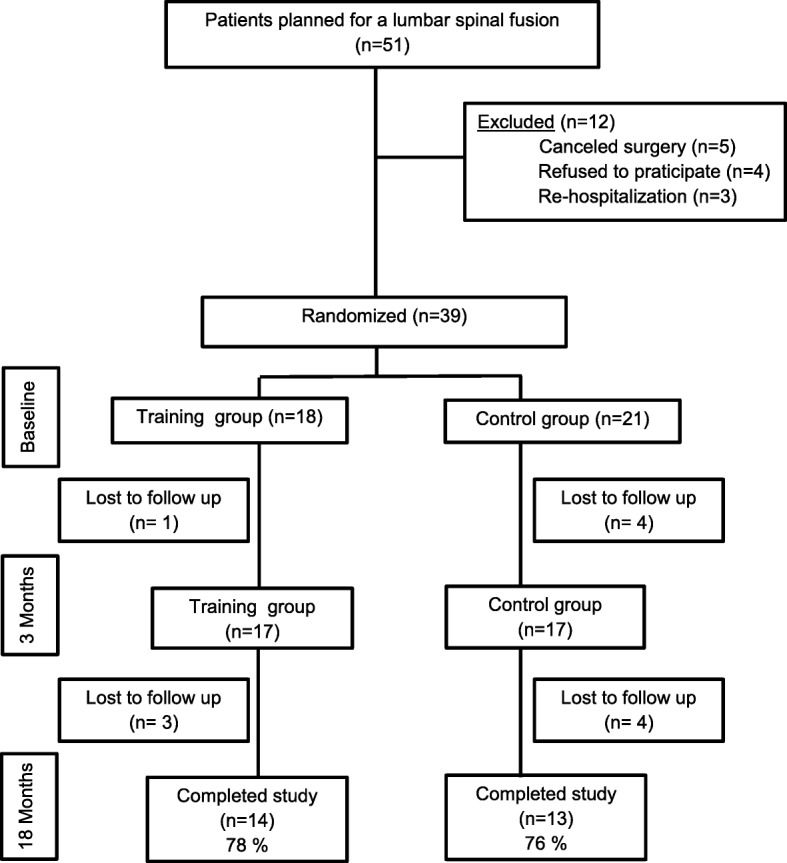
Consent subject flow diagram

All subjects received written and verbal informed consent information regarding their participation in the rehabilitation program. Subjects were required to provide signed informed consent and complete questionnaires. The method of concealed random allocation without blocking was used to form groups. As shown in a consent flow diagram (Fig. 1), 51 subjects planned for elective LSF fulfilled the study’s inclusion criteria. A total of 12 subjects were excluded from the study: five due to surgery exceeding the inclusion criteria (decompression of two or more levels in four subjects, two-level fusion in one subject), four refused inclusion, one due to postoperative infection, and two due to re-hospitalization for unrelated causes. Five subjects were lost during the training period and an additional seven at latest follow-up.

By random allocation, the training group included 36% male subjects, age 60.3 (SD ± 8.1), and body mass index 27.7 (SD ± 2.7), and the control group 69% male subjects, age 61.1 (SD ± 8.1), and body mass index 30.2 (SD ± 5.6) (Table 1). For the power calculation, the ODI was used. Based on earlier studies, the standard deviation was set to 10 points [16]. A 14.1-point difference in this category was considered clinically significant. Assuming a power = 80%, a total of 32 subjects were required. For the training period, the recommended power was achieved but not for the latest follow-up.

**Table 1:** Background subject data

Subject characteristics	Control group (*n* = 14)	Training group (*n* = 13)
Male (%)	36	69
Age (year ± SD)	60.3 ± 8.1	61.1 ± 8
Body mass index (kg/m^2^ ± SD)	27.7 ± 2.7	30.2 ± 5.6

### Control group

The control group followed the hospital’s standard protocols. These did not include exercises or physiotherapy prior to 3 months postoperatively.

### Training group

The training group performed the rehabilitation program twice per week over 9 weeks, starting 3 weeks after the surgery. During the first training sub-period (week 1 to week 5), isometric exercises were focused on the trunk extension, flexion, and lateral flexion muscles by maintaining the lumbar spine’s neutral position. Each exercise was maintained for 15 s initially, separated by 45 s’ rest, and repeated three times. After each training protocol, the subjects were asked to assess the level of intensity on a 10-point Borg scale. When the level of perceived effort felt below 8, they increased the exercise duration to 20, 25, and 30 s. Interferential electrical current therapy of the trunk extensor muscles in the lumbar region, lasting 20 min, and with a frequency of 5 Hz was applied in that sub-period. During the period from week 6 to week 9, the exercises were performed with strength machines and the duration extended to 30 s. Leg adduction and hip extension exercises were added. The subjects were instructed to increase IAP with co-activation of the abdominal muscles (abdominal bracing) and maintain the neutral position of their lumbar spine before and during the exercises. Following strength training, static stretching was applied to the exercised muscle. The training sessions were supervised by the same physiotherapist.

### Evaluation of the functional outcome

For each participating subject, the following background data were registered:

• Isometric trunk muscle strength

The isometric trunk muscle extension, flexion, and lateral flexion strength were measured using a strain-gauge dynamometer (Steinberg Systems, Poland). The measurements were performed in a standing position with the pelvis supported [17]. A belt was fastened around the upper body at shoulder level. Subjects gradually applied the maximal force and sustained it for 2–3 s*.* Maximum torque was calculated from the force sensor data and the lever as the distance between the middle line of the belt and the iliac crest level. Three maximal efforts were performed.

• Subjects’ physical performance

The physical parameters were as follows: walked distance during the 6-min walking test (6MWT) [18], number of stand-ups in 30 s during the chair stand test (CST) [19], and standing reach height test (SRH) [20].

• The IAP pre-activation pattern

To determine the initiation point of the IAP, abdominal lateral force was measured. The subjects stood in an upright position and pushed against the force plate. Time delay between the onset of lateral abdominal force rise and the onset of force rise of the force plate was calculated. Mechanical measurements were utilized rather than electromyography of m. transversus abdominis due to the confounding effect that too high skin fold may have on electromyography measurements.

• Subjects’ subjective self-evaluations

○ The Oswestry disability index (ODI) presented subjects with a score index from 0 to 100 where lower scores represent lower levels of low back pain disability [14, 15].

○ The visual analogue scale (VAS) presented subjects with a back pain intensity score index from 0 to 10, where 0 = “no problems” and 10 = “maximum problems” [16].

All parameters were measured at 3 weeks and 3 months following LSF. At 18 months postoperatively, all parameters, excluding IAP pre-activation pattern, were measured.

### Statistical analysis

An independent *t* test and Wilcoxon-Mann-Whitney *U* tests were applied to the data using the SPSS 20.0 for Windows. The risk of type 1 error was set to 5% (a significance level of 0.05). A one-way repeated-measures ANOVA was used with the functional outcome variable as the within-subject variable and the group variable as the between-subjects factor. The Bonferroni post hoc tests were used. Spearman’s rho was used to determine the correlation level between the functional outcome variables.

## Results

The training group subjects (14/14) had no fusion-related complications. Two subjects out of 13 in the control group had non-union problems and were excluded from the analysis. One subject experienced no pain, despite segmental movement shown in flexion/extension films, and was treated conservatively. The other was reoperated 2 months after index surgery due to overt hardware loosening and mechanical back pain.

**Fig. 2:**
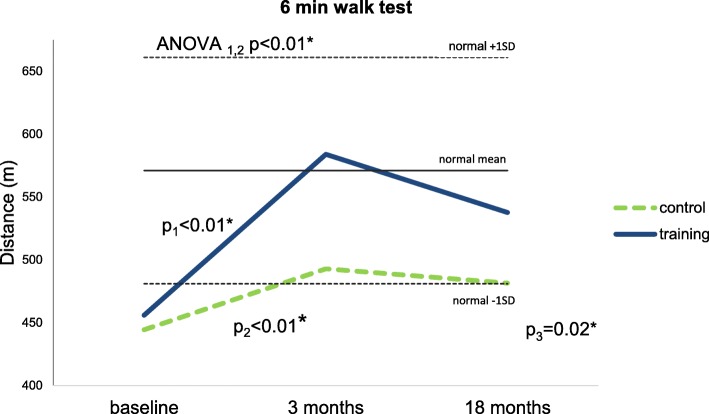
Six-minute walking test (6MWT) (group mean). *p*_1_—difference between the baseline’s and 3 months’ mean for the training group. *p*_2_—difference between the 3 months’ and 18 months’ mean for the control group. *p*_3_—difference between the baseline’s and 18 months’ mean for the training group. ANOVA_1,2_—one-way repeated-measures ANOVA between the baseline’s and 3 months’ mean. Horizontal solid and dashed lines indicate the mean expected walking distance (571 m), with 95% CI (± 90 m) in a normal, age-matched population, respectively [21]

Analysis of baseline data showed no statistically significant difference in 6MWT between the two groups. At 3 months postoperatively, a statistically significant training effect (*p* < 0.05) and improvement for both groups were observed. No statistically significant improvement was detected for either group at 18 months follow-up; however, the training group exceeded the expected walking distance (571 m, ± 90) when compared to age correlated normal [21]. This effect was seen to remain 18 months thereafter (Fig. 2).

**Fig. 3:**
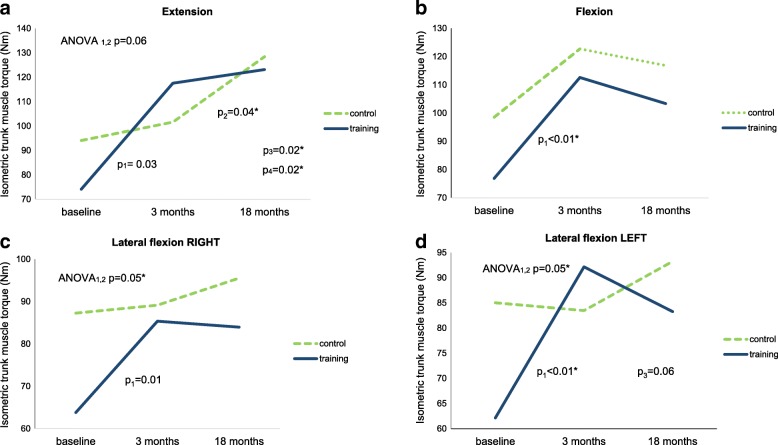
Isometric trunk muscle strength (Nm) (group mean). p_1_—difference between the baseline’s and 3 months’ mean for the training group. p_2_—difference between the 3 months’ and 18 months’ mean for the control group. p_3_—difference between the baseline’s and 18 months’ mean for the training group. p_4_—difference between the baseline’s and 18 months’ mean for the control group. ANOVA_1,2_—one-way repeated-measures ANOVA between the baseline’s and 3 months’ mean

Mean scores for the isometric trunk muscles strength, significant changes of differences between groups, and differences between means for each group are presented in Fig. 3. The training group significantly improved in all outcome measurements. A one-way repeated-measures ANOVA showed a significant training effect in both lateral flexions (both *p* = 0.05). In trunk extension, a tendency toward a significant improvement due to training was observed (*p* = 0.06). After 18 months, no training effects were observed in any of the trunk strength parameters.

No statistically significant differences between the groups in the progress of CST were observed after 3 and 18 months (Table 2). The training group demonstrated an initial improvement in SRH (*p* < 0.04); however, this gain was seen to reduce at further follow-up time points (*p* < 0.02). Time shift off the start with increase of IAP approached statistical significance within the training group (*p* < 0.08). ODI was reduced significantly in both groups after 3 months (both *p* < 0.001) and stayed at similar level after 18 months. VAS stayed at similar level during the follow-up time.

**Table 2:** Effect of rehabilitation on physical performance, pre-activation pattern and subjective self-evaluations

	Training group (*n* = 14)						Control group (*n* = 13)								
	Baseline	3 months	18 months	*P*_1_ value	*P*_2_ value	*P*_3_ value	Baseline	3 months	18 months	*P*_1_ value	*P*_2_ value	*P*_3_ value	ANOVA _1,2_ *P* value	ANOVA _2,3_ *P* value	ANOVA _1,3_ *P* value
Physical performance															
CST (repetitions)	11.5 (± 3.9)	17.1 (± 4.7)	17.6 (± 5.8)	0.00*	1.0	0.00*	10.5 (± 4.5)	14.7 (± 5.7)	15.3 (± 6.3)	0.00*	1.0	0.01*	0.3	0.9	0.4
SRH (cm)	210.4 (± 12.2)	213.6 (± 13.2)	210 (± 13.2)	0.02*	0.02*	1.0	212.2 (± 14.1)	211.8 (± 15)	212.5.8 (± 14.2)	1.0	1.0	1.0	0.04*	0.02*	0.6
The IAP pre-activation pattern															
Time before prime mover (s)	0.22 (± 0.42)	− 0.03 (±0.22)	/	0.04*	/	/	0.22 (± 0.25)	0.20 (± 0.18)	/	0.67	/	/	0.08	/	/
Subjective self-evaluations															
ODI	40.6 (± 11.9)	27.4 (± 13.4)	25.7 (± 15.1)	0.00*	1.0	0.02*	41.5 (± 6.1)	29.5 (± 12)	27.6 (± 22)	0.00*	1.0	0.04*	0.7	0.9	0.9
VAS	2.7 (± 1)	2.7 (± 1.3)	4 (± 2.3)	1.0	0.1	0.1	3.6 (± 1.5)	3.2 (± 1.2)	3.6 (± 2.6)	1.0	0.6	1.0	0.6	0.4	0.3

Data are reported as mean (SD)

*CST*, chair stand test, number of stand ups; *SHR*, standing reach height, numbers represent centimetres; *ODI*, Oswestry disability index, scoring from 0 to 100, 0 = no pain; *VAS*, visual analogue scale, scoring from 0 to 10, 0 = “no problems” and 10 = “maximum problems”; *Time before prime mover*, negative numbers represent time (s) before prime mover, positive represent time (s) after prime mover; *P*_*1*_*-value*, difference between the baseline’s and 3 months’ mean; *P*_*2*_ *value*, difference between the 3 months’ and 18 months’ mean; *P*_*3*_ value, difference between the baseline’s and 18 months’ mean; *ANOVA*, one-way repeated-measure ANOVA; *ANOVA*_*1,2*_ difference between the baseline’s and 3 months’ mean; *ANOVA*_*2,3*_ difference between the 3 months’ and 18 months’ mean; *ANOVA*_*1,3*_ difference between the baseline’s and 18 months’ mean

*Significance difference between the groups, *p* < 0.05

Correlation analysis showed that relative changes of extension and both lateral flexions were significantly correlated among themselves due to the training as well as the follow-up (Table 3). In trunk flexion, a tendency toward a significant correlation was observed (*p* = 0.06–0.08). A correlation between the CST and all isometric trunk muscle strength variables was found.

**Table 3:** Correlation between initial status and changes due to training in the training group

		Changes									
		6 MWT	CST	SRH	Flexion	LFR	LFL	Extension	ODI	VAS	TIME
Initial status	6 MWT	-0.81^*^	-0.42	0.08	-0.06	0.14	0.15	-0.06	0.25	0.26	-0.37
	CST	-0.09	-0.48	-0.29	-0.04	-0.28	-0.19	-0.33	0.27	0.32	-0.15
	SRH	-0.22	-0.46	0.02	-0.37	0.01	-0.01	-0.02	-0.38	-0.34	-0.41
	Flexion	-0.26	-0.65^*^	-0.07	-0.53^*^	-0.48	-0.51	-0.51	-0.36	-0.29	-0.44
	LFR	-0.21	-0.59^*^	0.03	-0.51	-0.59^*^	-0.65^*^	-0.53^*^	-0.33	-0.13	-0.30
	LFL	-0.28	-0.55^*^	-0.03	-0.45	-0.56^*^	-0.64^*^	-0.52	-0.32	-0.09	-0.42
	Extension	-0.19	-0.69^*^	0.03	-0.46	-0.56^*^	-0.56^*^	-0.54^*^	-0.34	-0.18	-0.34
	ODI	0.29	-0.05	0.25	0.01	0.06	0.14	0.06	0.08	0.12	0.23
	VAS	0.13	-0.14	0.43	-0.10	0.12	0.42	0.07	-0.24	-0.45	-0.09
	TIME	0.33	-0.04	0.20	-0.03	-0.32	-0.26	-0.23	-0.28	0.11	0.11

*6MWT* 6-minute walk test; *CST* chair stand test; *SHR* standing reach height; *LFR* lateral flexion right; *LFL* lateral flexion left; *ODI* Oswestry disability index; *VAS* visual analogue scale; *TIME* time before prime mover

^*^Significance difference, p< 0.05

## Discussion

The goal of the present study was to analyze the safety and effects of early initiation of a postoperative rehabilitation program based on strength training principles supporting IAP utilization for trunk stabilization after LSF. Both groups improved their walking distance after 3 months; however, only the training group achieved and maintained normal age correlated walking distance. The training group significantly improved trunk extension, lateral flexions on both sides, SRH, and pre-activation pattern, while the control group showed no such significant improvements. A similar improvement in trunk flexion and CST was observed for both groups. Subjective self-evaluations showed a similar improvement in ODI but no changes in pain for either group. No hardware loosening or failure was observed in the training group despite commencement of rehabilitation only 3 weeks after surgery.

On analysis of early initiation of postoperative rehabilitation, Oestergaard et al. failed to show any advantages after 6 months of rehabilitation with ODI, leading the authors to conclude that starting rehabilitation early may not be advantageous for LSF patients [14]. In the present study, an earlier start point of intervention was employed. This saw both groups display improvement with ODI after only 3 months of rehabilitation. It would therefore appear that early initiation of strength training based rehabilitation does not pose any adverse effect, yet early intervention does not result in significant gain in overall rehabilitation outcomes as both groups were seen to make similar progress.

However, on examination of the functional test results of the present study, the data showed that the strength training group demonstrated superior functional gains when compared to the standard rehabilitation group. An example of such functional gains would be walking, an important everyday functional task which has previously been shown to be impaired in low-back pain patients [22, 23]. The training group exceeded the normal population’s mean (571 ± 90 m), while the control group stayed beyond the area of one standard deviation [21]. The control group results are comparable to the 1-year follow-up results of Oestergaard et al. [8]. Improvement in the training group can be considered as clinically relevant, this was not shown to be the case for the control group [24]. This would lead us to conclude that early initiation of rehabilitation may not adversely affect a patient’s walking ability, as stated by Oestergaard et al. [8]. We acknowledge that a limitation of our study is the relatively small number of patients which could add to an overestimation of positive results.

Core muscle strength evaluated by trunk extension and lateral flexion was shown to be significantly improved in the training but not in the control group, whereas trunk flexion was found to be similarly improved for both groups. One possible explanation is fear of pain during trunk extension in the control group, which had no specific training to test their pain level during maximal effort [25]. Trunk extension torques were quite low in the present study compared to some other studies involving low-back pain [26]. Even after training, they remained substantially lower than in the Kienbacher et al. study [26], where trunk extension performance was supervised by a clinical psychologist to overcome any fear-related inhibition. Therefore, the results of the trunk extension test in the present study, where no specific fear control was introduced, may be attributed mainly to improved neuromuscular function, and partly to reduced fear-related inhibition.

One important aspect included in the present strength training based rehabilitation was the use of intra-abdominal pressure [9–11]. The subjects learned to use IAP during all exercises and were advised to also do so in daily life. To support this inclusion, the activation of abdominal muscles related to IAP showed that subjects from the training group systematically shifted the initiation of IAP before starting the action and therefore afforded better protection to their lumbar spine.

Lower initial level in a specific test resulted in a greater improvement in that test after training. However, the core strength tests represented a group where subjects with the lowest general core strength improved in all these tests the most. This finding would indicate that subjects most in need of improvement would obtain it to a greater degree. The changes observed in ODI and functional tests such as 6MWT or SRH were not shown to be related to the initial core strength level. CST was the only parameter demonstrating correlation between initial core strength and an improvement in a functional test. Thus, it may be concluded that the subjects’ initial functional and strength level had an important effect on the training outcome.

The functional advantage of the training group after 3 months of rehabilitation was mostly lost at the end of the follow-up. If we assume that this plateau in functional gain represents a return to normal daily functional ability, then the data shows that 2 months engagement in the current training strategy is sufficient to achieve or even exceed normal functional performance.

## Conclusions

The present study showed that early initiation of a postoperative rehabilitation program based on strength training principles supporting IAP utilization for trunk stabilization after LSF is safe and effective and enable earlier functional recovery than standard rehabilitation protocol.

## Abbreviations

6MWT: 6-min walking test
CST: Chair stand test
IAP: Intra-abdominal pressure
LSF: Lumbar spine fusion
ODI: Oswestry disability index
SRH: Standing reach height test
